# Sociodemographic, Behavioral, and Psychosocial Factors Associated With Mammography Screening Uptake Among Women in Saudi Arabia: A Cross-Sectional Survey

**DOI:** 10.7759/cureus.85785

**Published:** 2025-06-11

**Authors:** Nada R Alharbi, Nouf M Almuhaimel, Fayruz A Almansouri, Nourah E Alsaiari, Rania A Alshammari, Nura N Alahmadi, Mohd H Yusuf

**Affiliations:** 1 General Surgery, King Salman Specialist Hospital, Hail, SAU; 2 Radiology, King Fahd Hospital, Medina, SAU; 3 General Practice, Salmaniya Medical Complex, Manama, BHR

**Keywords:** barriers, breast cancer, health behavior, mammography, saudi arabia, screening uptake

## Abstract

Background: Breast cancer is a leading cause of morbidity and mortality among women worldwide. Despite free mammography screening availability in Saudi Arabia, participation rates remain low. This study aimed to identify sociodemographic, behavioral, and psychosocial factors associated with mammography screening uptake among women in Saudi Arabia.

Methodology: A cross-sectional survey was conducted from March to April 2025, involving 487 women aged 18 years and older across Saudi Arabia. Participants completed a questionnaire assessing demographics, health behaviors, breast cancer awareness, and barriers to screening. The primary outcome was self-reported mammogram uptake. Statistical analysis included bivariate tests and multivariable logistic regression.

Results: Out of 487 participants, 169 (34.7%) reported having undergone a mammogram. Screening uptake was higher among women aged ≥40 years (142/267; 53.2%) compared to those <40 years (27/220; 12.3%). Women with a family history of breast cancer were more likely to have been screened (74/121; 61.2%) than those without (95/366; 26.0%). Similarly, 58/97 (59.8%) women performing monthly breast self-examinations reported screening, compared to 111/390 (28.5%) who did not. Fear of cancer detection was reported by 154 participants (31.6%) and was associated with lower screening uptake (39/154, 25.3%) versus those without fear (130/333, 39.0%). Multivariable analysis confirmed age ≥40 years (adjusted odds ratio (aOR) 2.61, *P *< 0.001), family history (aOR 2.23, *P *< 0.001), and monthly self-examination (aOR 1.79, *P *= 0.019) as positive predictors, while fear of diagnosis was negatively associated (aOR 0.56, *P *= 0.014).

Conclusions: Mammography uptake among women in Saudi Arabia remains suboptimal. Older age, family history, and proactive health behaviors increase screening likelihood, while fear of diagnosis reduces it. Targeted interventions addressing emotional and educational barriers are essential to enhance participation.

## Introduction

Breast cancer remains the most frequently diagnosed cancer and a leading cause of cancer-related death among women worldwide [1]. Early detection through organized screening programs, primarily mammography, has been proven to reduce breast cancer mortality by identifying tumors at earlier, more treatable stages [1,2]. International guidelines generally recommend routine mammographic screening for women aged 40 years and older, tailored according to individual risk profiles [3-5].

In Saudi Arabia, breast cancer represents the most common malignancy in women, accounting for approximately 29% of all female cancers and showing a rising incidence over recent decades [2,4]. Despite improvements in healthcare infrastructure and the availability of free mammography screening through the national healthcare system, uptake remains low, with studies reporting rates between 10% and 35% [2-4]. This discrepancy between availability and utilization highlights ongoing barriers to effective screening implementation.

Several factors have been implicated in limiting breast cancer screening uptake in the Saudi context. These include limited knowledge and awareness about breast cancer and screening guidelines, cultural and religious beliefs that may discourage seeking preventive care, fear of cancer diagnosis, social stigma, and logistical obstacles such as transportation and accessibility [6,7]. Furthermore, previous research has suggested that women’s perceptions about susceptibility and benefits of screening, as well as their health-seeking behaviors, play crucial roles in screening decisions.

While prior studies have investigated knowledge and attitudes towards breast cancer screening in Saudi Arabia, there remains a paucity of data examining the multifactorial determinants, including sociodemographic, behavioral, and psychosocial factors influence actual screening uptake [8-11]. A comprehensive understanding of these factors is essential for developing targeted interventions that address the specific barriers faced by Saudi women and improve screening rates.

This study aims to assess the factors associated with mammography screening uptake among women in Saudi Arabia, using a survey-based approach to capture relevant demographic, behavioral, and psychosocial variables. The findings will inform policymakers and healthcare providers seeking to optimize breast cancer screening strategies in this population.

## Materials and methods

Study design and setting

We conducted a cross-sectional, survey-based study between March 1, 2025, and April 31, 2025, in Saudi Arabia to evaluate factors associated with breast cancer screening uptake among adult women. The study was conducted across various urban and semi-urban communities through both in-person and online data collection to ensure broad representation.

Study population and sampling

Eligible participants were women aged 18 years or older residing in Saudi Arabia, regardless of nationality, who were able to read and respond to the questionnaire in either Arabic or English. Women with a personal history of breast cancer were excluded to avoid confounding due to diagnostic rather than screening-related mammography.

A convenience sampling strategy was employed, recruiting participants through primary healthcare centers, community outreach programs, social media platforms, and university networks. To improve demographic diversity, recruitment efforts targeted multiple regions across the country.

A minimum sample size of 385 was calculated using a single proportion formula with a 95% confidence level, an expected screening uptake of 50% (to maximize sample size), and a 5% margin of error. Accounting for nonresponse and incomplete data, the final target was set at ≥450 participants.

While our sample included diverse regions and age groups, no formal comparison to national demographic statistics was performed. Future studies should include such comparisons to enhance external validity and contextualize findings.

Questionnaire development

The structured questionnaire was developed in English and translated into Arabic using a forward-backward translation method by two independent bilingual translators. Content validity was assessed by a panel of three experts in public health and radiology. A pilot test was conducted on 25 participants (excluded from the final analysis) to evaluate clarity, language appropriateness, and time to completion. Minor modifications were made based on feedback.

The questionnaire consisted of 28 items across four domains. Most items used binary (yes/no) responses, while some knowledge and belief questions used a 3-point Likert scale (agree/neutral/disagree). The final questionnaire comprised four main sections. The first addressed sociodemographic data, including age, nationality, marital status, education level, and employment status. The second section covered health history, such as the presence of a family history of breast cancer and any prior breast cancer screening behavior. The third section focused on awareness and beliefs, assessing participants’ knowledge of breast cancer screening and their sources of information. The final section explored perceived barriers to screening, including concerns related to cost, fear of diagnosis, access to services, and cultural or societal factors. All questions were multiple choice, including both single-response and forced-choice formats (Appendix).

The primary outcome was self-reported mammogram uptake, defined as a binary response to the question: “Have you ever had a mammogram for breast cancer screening?” Participants who responded “yes” were classified as having undergone screening. The age threshold of ≥40 years was used to stratify analysis based on standard recommendations for breast cancer screening.

Data collection and statistical analysis

Data were collected anonymously using a combination of paper-based forms and secure online forms (Google Forms). Informed consent was obtained electronically or in writing before participation. No personally identifiable data were collected. Data were entered into and analyzed using IBM SPSS Statistics version 27 (IBM Corp., Armonk, NY). Descriptive statistics were used to summarize participant characteristics, with frequencies and percentages reported for categorical variables and means with standard deviations for continuous variables.

Bivariate analyses were conducted using chi-square tests (or Fisher’s exact test, where appropriate) to assess associations between independent variables and mammogram uptake. Variables with *P *< 0.10 in bivariate analyses were considered for inclusion in a multivariable logistic regression model to identify independent predictors of screening uptake. Adjusted odds ratios (aORs) with 95% confidence intervals (CIs) and corresponding *P*-values were reported. A two-tailed *P*-value < 0.05 was considered statistically significant.

Multicollinearity was assessed using variance inflation factors (VIFs), and no concerning multicollinearity was detected (VIF < 2.0 for all predictors). Categorical variables were dummy coded, with reference categories noted in the model output. Assumptions for logistic regression, including linearity in the logit for continuous predictors, were assessed and met.

## Results

Participant characteristics

A total of 487 women completed the questionnaire. Of the 487 completed questionnaires, 312 (64.1%) were collected online and 175 (35.9%) were collected in person. The majority were Saudi nationals (456 of 487, 93.7%). The largest age group was 40-49 years (141 of 487, 29.0%), followed by 30-39 years (126, 25.9%) and <30 years (94, 19.3%). A smaller proportion were aged 50-59 years (78, 16.0%) or ≥60 years (48, 9.9%). Regarding education, 197 participants (40.5%) held a university degree and 77 (15.8%) had postgraduate education, while 152 (31.2%) had completed high school (Table 1).

**Table 1:** Sociodemographic characteristics of the participants (N = 487). Percentages may not total 100 because of rounding. Age group, nationality, and education level were self-reported. No formal education was defined as the absence of any school-based instruction.

Characteristics		n	%
Age group (years)	<30	94	19.3%
	30-39	126	25.9%
	40-49	141	29.0%
	50-59	78	16.0%
	≥60	48	9.9%
Nationality	Saudi	456	93.7%
	Non-Saudi	31	6.3%
Education level	No formal education	18	3.7%
	Primary	43	8.8%
	High school	152	31.2%
	University	197	40.5%
	Postgraduate	77	15.8%

Screening knowledge and behavior

Of the total, 169 women (34.7%) reported having undergone a mammogram at least once. Breast self-examination every month was reported by 97 participants (19.9%). A family history of breast cancer was present in 121 respondents (24.8%). Prior education or counseling about breast cancer screening was reported by 318 women (65.3%). A total of 154 participants (31.6%) expressed fear of discovering cancer as a barrier to screening, while 413 (84.8%) believed that early detection improves survival (Table 2).

**Table 2:** Breast cancer screening knowledge and behavior (N = 487). Values are frequencies and percentages based on participant self-report. Percentages may not total 100 because of rounding. Prior education refers to any formal or informal session or material related to breast cancer awareness.

Variable	n	%
Ever had a mammogram	169	34.7%
Performs monthly breast self-examination	97	19.9%
Family history of breast cancer	121	24.8%
Received prior education on breast cancer	318	65.3%
Afraid of cancer detection via screening	154	31.6%
Believes screening improves survival	413	84.8%

Bivariate associations with screening uptake

Mammogram uptake was significantly more common among women aged 40 years or older (142 of 267, 53.2%) compared to those under 40 (27 of 220, 12.3%; *P *< 0.001). Uptake was also higher among those with a university or postgraduate degree (111 of 274, 40.5%) versus lower education levels (58 of 213, 27.2%; *P *= 0.008). Women with a family history of breast cancer were more likely to have been screened (74 of 121, 61.2%, vs. 95 of 366, 26.0%; *P *< 0.001). Prior exposure to breast cancer education (133 of 318, 41.8%, vs. 36 of 169, 21.3%; *P *= 0.003) and performing monthly self-examinations (58 of 97, 59.8%, vs. 111 of 390, 28.5%; *P *< 0.001) were also associated with higher screening uptake. In contrast, fear of discovering cancer was associated with lower uptake (39 of 154, 25.3%, vs. 130 of 333, 39.0%; *P *= 0.014). Belief in the benefit of screening was not significantly associated with screening behavior (156 of 413, 37.8%, vs. 13 of 74, 17.6%; *P *= 0.457) (Table 3).

**Table 3:** Bivariate associations with mammogram uptake (n = 487). Values represent the number and percentage of participants who reported ever having a mammogram within each subgroup. *P*-values were calculated using the chi-square test. A *P*-value of less than 0.05 was considered statistically significant. All variables were self-reported.

Variable	Had mammogram, *n*/*N* (%)	*P*-value
Age ≥ 40 years	142/267 (53.2%)	<0.001
University or higher education	111/274 (40.5%)	0.008
Family history of breast cancer	74/121 (61.2%)	<0.001
Received screening education	133/318 (41.8%)	0.003
Afraid of cancer detection	39/154 (25.3%)	0.014
Performs self-exams monthly	58/97 (59.8%)	<0.001
Believes screening is beneficial	156/413 (37.8%)	0.457

Multivariable analysis

In the logistic regression model (Table 4), age ≥40 years was independently associated with increased odds of having undergone a mammogram (aOR 2.61; 95% CI, 1.68-4.05; *P *< 0.001), as was a family history of breast cancer (aOR 2.23; 95% CI, 1.45-3.42; *P *< 0.001). Monthly self-examination remained a significant predictor (aOR 1.79; 95% CI, 1.10-2.91; *P *= 0.019). Fear of cancer detection was inversely associated with screening (aOR 0.56; 95% CI, 0.36-0.89; *P *= 0.014). The association between prior breast cancer education and screening did not remain significant in the adjusted model (aOR 1.34; 95% CI, 0.88-2.06; *P *= 0.170).

**Table 4:** Multivariable logistic regression analysis of factors associated with mammogram uptake. Adjusted odds ratios (ORs), 95% confidence intervals (CIs), and *P*-values were derived from a logistic regression model. The model included all listed variables. An OR greater than 1 indicates higher odds of having had a mammogram; an OR less than 1 indicates lower odds. A *P*-value of less than 0.05 was considered statistically significant.

Variable	Adjusted OR	95% CI	*P*-value
Age ≥ 40 years	2.61	1.68-4.05	<0.001
Family history of breast cancer	2.23	1.45-3.42	<0.001
Monthly self-exam	1.79	1.10–2.91	0.019
Afraid of cancer detection	0.56	0.36-0.89	0.014
Received screening education	1.34	0.88-2.06	0.170

## Discussion

In this cross-sectional study of 487 women in Saudi Arabia, we found that only 169 participants (34.7%) had ever undergone a mammogram, despite the majority being age-eligible for screening and aware of its potential benefits. Uptake was significantly higher among women aged ≥40 years, those with a family history of breast cancer, and those who performed regular breast self-examinations. Conversely, fear of discovering cancer was independently associated with lower screening rates.

These findings align with previous studies in the Gulf region and other middle-income countries, which have consistently shown suboptimal breast cancer screening uptake despite relatively high levels of awareness [10-14]. The strongest predictor in our model - age ≥40 years - is consistent with international screening guidelines, but the observed rate of screening among age-eligible women (53.2%) remains below the national target set by the Saudi Ministry of Health.

The positive association between family history and screening is well-established and likely reflects increased perceived susceptibility. Interestingly, monthly breast self-examination - a behavior often considered outdated as a screening tool - emerged as a strong independent correlate of mammogram uptake. This may reflect a broader health-seeking attitude among these women, as suggested by similar findings in regional studies [12-16].

Fear of discovering cancer was a significant barrier to screening, even after adjustment for other factors. This finding echoes longstanding concerns about emotional barriers in preventive care, particularly in conservative societies where cancer may still carry stigma [10,14]. Addressing such fears through culturally tailored education and provider counseling may be critical to improving screening rates.

Although prior exposure to breast cancer education was associated with higher uptake in bivariate analysis, this association did not remain significant after adjustment, suggesting that knowledge alone may not be sufficient to change behavior. This underscores the importance of multifactorial interventions that address not only awareness but also accessibility, emotional readiness, and structural barriers.

Strengths and limitations

This study has several strengths, including a relatively large and demographically diverse sample, the use of a validated bilingual questionnaire, and comprehensive multivariable analysis. It also contributes to the limited literature on breast cancer screening behavior in the Gulf region, particularly among non-clinical populations.

However, several limitations should be noted. First, the use of convenience sampling limits the generalizability of findings, and selection bias may have favored more health-conscious individuals. Second, screening status was self-reported and may be subject to recall or social desirability bias. Third, the cross-sectional design precludes causal inference, although observed associations align with existing literature. Additionally, some potentially important variables, such as health insurance status, proximity to healthcare facilities, and physician recommendation, were not assessed and may influence screening uptake. The mixed-method data collection approach (online and in-person) may have introduced bias related to digital literacy, access, or self-selection. Finally, although we examined constructs such as fear of cancer detection and prior education on screening, the full survey instrument and question phrasing were not included in the initial manuscript but have now been added to enhance clarity and support reproducibility (Appendix).

## Conclusions

In conclusion, breast cancer screening uptake among women in Saudi Arabia remains suboptimal despite available services and general awareness. Our findings identify age, family history, and proactive health behaviors as key facilitators of screening, while fear of cancer detection constitutes a significant barrier. These insights underscore the need for culturally sensitive, multifaceted interventions that address emotional and educational barriers, alongside improved access, to enhance screening participation and ultimately reduce breast cancer morbidity and mortality in this population.

While this study provides valuable insights into factors associated with mammography screening uptake among women in Saudi Arabia, the findings should be interpreted with caution. Due to the use of convenience sampling and potential underrepresentation of certain subgroups, such as women from rural areas or lower-income backgrounds, the results may not be fully generalizable to the entire national population. Although efforts were made to include a demographically diverse sample, future studies employing probability-based sampling methods are needed to validate and expand upon these findings in a more representative cohort.

## Appendices

Appendix 

**Table 5:** Survey questionnaire

Section	Question	Response Options
1. Sociodemographic Data	Age	<30, 30–39, 40–49, 50–59, ≥60
	Nationality	Saudi, Non-Saudi
	Marital status	Single, Married, Divorced, Widowed
	Education level	No formal education, Primary, High school, University, Postgraduate
	Employment status	Employed, Unemployed, Student, Retired
2. Health History	Do you have a family history of breast cancer?	Yes, No
	Do you perform breast self-examination monthly?	Yes, No
	Have you ever had a mammogram for breast cancer screening?	Yes, No
3. Awareness and Beliefs	Have you ever received any education or information about breast cancer screening?	Yes, No
	Do you believe breast cancer screening improves survival?	Agree, Neutral, Disagree
	Do you know the recommended age to start mammogram screening?	Open-ended or multiple choice
	What is your main source of information on breast cancer?	TV/Radio, Social media, Healthcare provider, Family/friends, Others
4. Perceived Barriers	Are you afraid of discovering you have cancer through screening?	Yes, No
	Is cost a barrier to getting a mammogram?	Yes, No
	Is transportation or distance to a facility a barrier?	Yes, No
	Do cultural or societal factors prevent you from undergoing screening?	Yes, No
	Do you feel embarrassed about the screening process?	Yes, No
	Have you ever been recommended by a physician to get a mammogram?	Yes, No
